# New biomolecular tools for aerobiological monitoring: Identification of major allergenic Poaceae species through fast real‐time PCR

**DOI:** 10.1002/ece3.3891

**Published:** 2018-03-25

**Authors:** Sofia Ghitarrini, Elisa Pierboni, Cristina Rondini, Emma Tedeschini, Gloria R. Tovo, Giuseppe Frenguelli, Emidio Albertini

**Affiliations:** ^1^ Department of Agricultural, Food and Environmental Sciences (DSA3) University of Perugia Perugia Italy; ^2^ Istituto Zooprofilattico Sperimentale dell'Umbria e delle Marche (IZSUM) Perugia Italy

**Keywords:** aerobiology, DNA, extraction, grasses, pollen, real‐time PCR

## Abstract

Grasses (Poaceae) are very common plants, which are widespread in all environments and urban areas. Despite their economical importance, they can represent a problem to humans due to their abundant production of allergenic pollen. Detailed information about the pollen season for these species is needed in order to plan adequate therapies and to warn allergic people about the risks they take in certain areas at certain moments. Moreover, precise identification of the causative species and their allergens is necessary when the patient is treated with allergen‐specific immunotherapy. The intrafamily morphological similarity of grass pollen grains makes it impossible to distinguish which particular species is present in the atmosphere at a given moment. This study aimed at developing new biomolecular tools to analyze aerobiological samples and identifying major allergenic Poaceae *taxa* at subfamily or species level, exploiting fast real‐time PCR. Protocols were tested for DNA extraction from pollen sampled with volumetric and gravimetric methods. A fragment of the *matK* plastidial gene was amplified and sequenced in Poaceae species known to have high allergological impact. Species‐ and subfamily‐specific primer–probe systems were designed and tested in fast real‐time PCRs to evaluate the presence of these *taxa* in aerobiological pollen samples. Species‐specific systems were obtained for four of five studied species. A primer–probe set was also proposed for the detection of Pooideae (a grass subfamily that includes also major cereal grains) in aerobiological samples, as this subfamily includes species carrying both grass allergens from groups 1 and 5. These, among the 11 groups in which grass pollen allergens are classified, are considered responsible for the most frequent and severe symptoms.

## 1 INTRODUCTION

Grasses (Poaceae) are ubiquitous plants, which are widespread in all environments and urban areas, representing about 20% of the world vegetation cover (Mabberley, 1987). This family counts about 780 genera and 12,000 species (Christenhusz & Byng, 2016), among which many cultivated cereals, that constitute the basis of human nutrition. Spontaneous species often occupy untamed fields, grazing lands but also public gardens and street borders. They are anemophilous plants, and due to their diffusion and abundant production of pollen, they often cause allergic reactions. Up to 40% of allergic individuals carry serum IgE antibodies reacting with grass pollen allergens and exhibit immediate‐type symptoms upon contact with grass pollen (Lockey & Bukantz, 1998). In Europe, where more than 420 species can be found (Emberlin, 1997; Galán, Cuevas, Infante, & Dominguez, 1989), grasses represent the first cause of pollinosis (D'Amato et al., 2007; De Weger et al., 2011). The highest prevalence occurs in young adults: 8%–35% of them in EEC countries have IgE serum antibodies to grass pollen (Burr, 1999; D'Amato, 2000). IgE‐associated allergic diseases, such as asthma and rhinitis, are recognized as a global health problem that is constantly increasing in severity (Bousquet et al., 2011). These conditions have been recognized to significantly reduce the quality of life and to have a serious economic impact on society (Bosque, Van Cauwenberge, & Khaltaev, 2001).

Several scientists have investigated the importance of grass pollen as outdoor aeroallergen, focusing on the mechanisms of patient sensitization and therapies (Durham et al., 1999; Moreira et al., 2015; Tripodi et al., 2012), relationship between grains/allergens concentration and symptoms (Annesi‐Maesano et al., 2012; Erbas et al., 2012; Feo Brito et al., 2010), patterns of emission (Emberlin et al., 2000; Fernández Rodríguez et al., 2014; Galán, Emberlin, Domínguez, Bryant, & Villamandos, 1995; Ghitarrini, Galán, Frenguelli, & Tedeschini, 2017; Sánchez‐Mesa et al., 2003), and influence of meteorological factors on the pollen season timing and intensity (García‐Mozo, Mestre, & Galán, 2010; Ghitarrini, Tedeschini, Timorato, & Frenguelli, 2017; Smith et al., 2009; Zhang et al., 2015). The grasses that mostly contribute to the airborne pollen load vary spatially, but those responsible for grass pollinosis are essentially included into a group of about 20 species, principally belonging to the subfamily Pooideae. This subfamily comprises also cultivated cereal species, such as wheat, barley, and corn, but these plants are known for not releasing relevant amounts of pollen in the atmosphere (e.g., cleistogamous species, or very big and heavy pollen grains). A minority of allergenic species of temperate and subtropical areas belong to subfamilies Chloridoideae, Panicoideae, Arundinoideae, and Bambusoideae (Weber, 2004). The allergenicity of Poaceae is linked to a limited number of proteins classified in 11 allergen groups, each one referring to molecules with similar physicochemical and immunological properties (Hrabina, Peltre, Van Ree, & Moingeon, 2008). Those identified in one species can often have homologous in others (Andersson & Lidholm, 2003; Ferreira, Hawranek, Gruber, Wopfner, & Mari, 2004; http://www.allergome.org) . While some grass allergens are shared across the entire vegetal kingdom or between pollinating plants, others are restricted to Poaceae (group 1) or to Pooideae (groups 2 and 5). From a molecular point of view, the subfamily Pooideae represents a very homogeneous group (Hrabina et al., 2008); their pollen can be considered the most harmful, as it contains group 1 and 5 allergens, that together account for more than 80% of grass pollen allergenicity, and as such are the most critical allergens for sensitization and desensitization processes (Frenguelli et al., 2010; Niederberger et al., 1998; Valenta et al., 1999; Van Ree, van Leeuwen, & Aalberse, 1998).

Given this complex background, allergic patients are often polyexposed and polysensitized to a mix of allergens that changes with the geographical zone (Peltre, 2007). Classic aerobiological monitoring has some limitations in the dissection of grass pollen season. The problem arises from the overlapping flowering periods of the different species, and on the extreme morphological similarity of their pollen grains (Perveen, 2006), which does not allow their discrimination at species level through microscope identification. In fact, during the routine analysis of aerobiological samples, it is not possible to establish the relative levels of the different species pollinating in a certain area. This information, instead, would be very useful in the diagnosis and therapy planning. The diagnosis of grass pollen allergy has benefited in recent years from the advent of purified recombinant allergens. This new approach, called component‐resolved diagnosis, allows the dissection of patient‐specific patterns of IgE reactivity to grass pollen allergen at molecular level (Jutel et al., 2005; Laffer et al., 1996; Mari, 2003; Valenta, Vrtala, Ebner, Kraft, & Scheiner, 1992), and consequently, the selection of exactly those allergens for specific immunotherapy to which the patient is sensitized (Ball et al., 1999). Despite the existence of a certain level of sequence homology and thus cross‐reactivity between grass allergens (Leiferman & Gleich, 1976), qualitative and quantitative differences exist between grass species. A significant source of heterogeneity arises from various protein isoforms originating from multiple genes, alternate splicing, or post‐transcriptional modifications (Hrabina et al., 2008). For these reasons, the dissection of the presence of the diverse grass pollen *taxa* in the atmosphere of a certain area would be crucial in modern and personalized medical approaches. Airborne allergen monitoring would also be an option (Chapman, 1998), but it requires additional and specific equipment (Plaza, Alcázar, Velasco‐Jiménez, & Galán, 2017).

Biomolecular techniques for the identification of organisms at various levels have become extremely common and relatively easy to apply. Over the past decade, the power of DNA barcoding has opened up new fields in taxonomic, ecological, and evolutionary research by facilitating species identification (Bell et al., 2016), and recently, such approaches have been applied in the identification of plants based on their pollen. Pollen samples collected from honeybees, bee nests, and honey have been analyzed with barcoding and metabarcoding approaches in a number of studies, using both nuclear (e.g., ITS2; Keller et al., 2015; Richardson, Lin, Sponsler, et al., 2015; Sickel et al., 2015;. *actin*; Torricelli, Pierboni, Tovo, Curcio, & Rondini, 2016) and plastidial (e.g., *rbcL*,* trnL*,* matK*) (Bruni et al., 2015; Galimberti et al., 2014; Hawkins et al., 2015; Richardson, Lin, Quijia, et al., 2015; Valentini, Miquel, & Taberlet, 2010) marker regions. Pollen DNA extraction and downstream processing are less common with aerobiological samples, which contain a small but very complex amount of biologic material. Few researchers have worked on these issues; Longhi et al. (2009) conducted a preliminary investigation on the possibility to substitute microscopic counts with quantitative PCR (qPCR) analysis, but did not test the method on real samples. Kraaijeveld et al. (2015) developed an efficient protocol to identify and quantify pollen in mixed samples, based on next‐generation sequencing. Nevertheless, such approach is not likely to be routinely applied, due to the time and costs needed to obtain and analyze metabarcoding sequencing data. Mohanty, Buchheim, and Levetin (2017) designed specific primers to be used in qPCR for a quick evaluation of the differential presence of *Juniperus* species in aerobiological samples.

The aim of this study was to assess a simple biomolecular procedure, applicable on routine basis, to identify grass species in aerobiological samples with higher resolution respect to microscope pollen counts. We identified a subset of Poaceae species among the most widespread in the area of Perugia (Central Italy) (Ghitarrini, Tedeschini, et al., 2017), all belonging to the Pooideae subfamily and highly allergenic, and exploited their DNA sequence to design fast real‐time PCR protocols to verify their presence in different types of aerobiological real samples. The application of such techniques could integrate classic aerobiological monitoring data and help the “decomposition” of the cumulative grass pollination curve that usually represents a sum of the pollens released contemporarily by different species, which cannot be discriminated.

## 2 MATERIALS AND METHODS

### 2.1 Airborne pollen sampling

Routine pollen sampling has been continuously carried out in Perugia since 1982, using a volumetric Hirst‐type 7‐day spore trap (Hirst, 1952) VPPS 2000 (Lanzoni, Bologna, Italy) placed on the roof of the Department of Agricultural, Food and Environmental Sciences (DSA3—University of Perugia) at about 20 meters above ground level. Special airborne pollen sampling took place from 18 April 2016 to 18 September 2016—covering the main grass pollen season in the area of the study—by an identical volumetric sampler, placed on a terrace, about 10 meters away from the first one. As indicated in the Minimum Requirements published by Galán et al. (2014) and Jäger et al. (1995), for both of the traps, the sampling support consisted in a plastic tape (melinex) brushed with an adhesive silicon (polydimethylsiloxane) (Lanzoni, Bologna, Italy), on which the pollen grains stick during the sampling. Samples from the first trap were prepared following the guidelines of the European Aerobiology Society Working Group (Galán et al., 2014) and used to perform traditional microscopic pollen counts as routine. The main parameters describing the grass pollen season (start, end, duration, peak day, annual pollen integral—API) were analyzed. The 5% threshold method (Nilsson & Persson, 1981) was used to calculate the start and end dates of the period of maximum pollen emission.

Melinex tapes from the second sampler were processed in various ways in order to extract DNA from the pollen stuck on its surface.

A Petri dish of 35 mm of diameter, with the bottom spread with silicon, was exposed on a specially built support, near the volumetric samplers, in order to catch pollen by gravimetric deposition. It was sheltered from the rain by a small canopy placed about 30 cm above the surface, thus leaving the air circulate freely over the samples. The dish was replaced weekly.

### 2.2 Plant material collection

**Figure 1:**
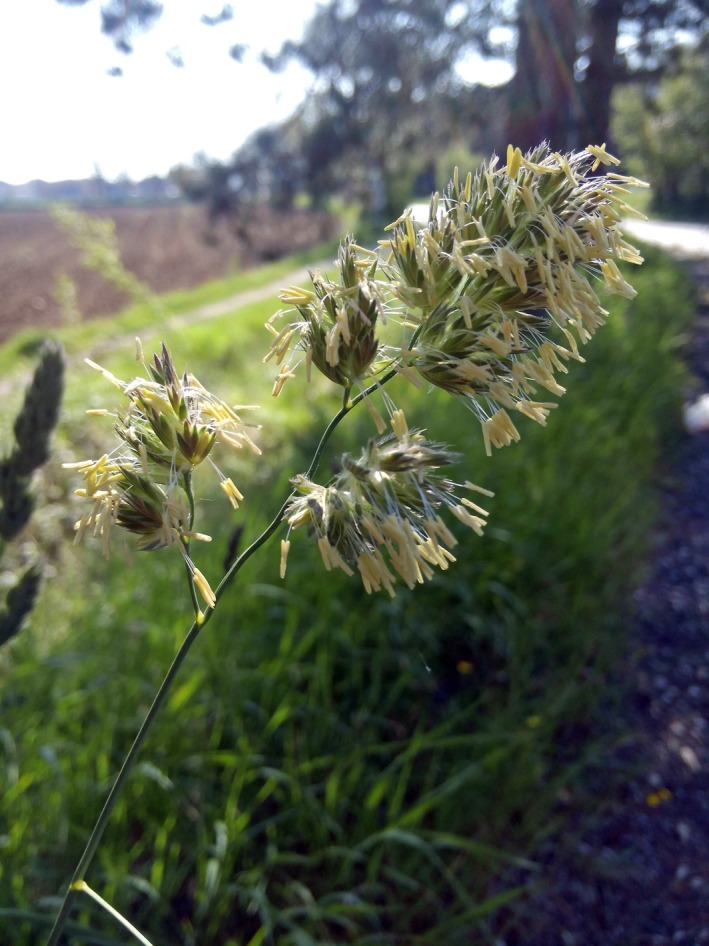
*Dactylis glomerata* panicle in full pollination (Perugia‐Italy, spring 2015)

Species to be investigated were selected based on the information on their richness in allergens (Hrabina et al., 2008) and on their distribution in the area of Perugia (Frenguelli et al., 2010; Ghitarrini, Tedeschini, et al., 2017). They were as follows: *Dactylis glomerata* L. (orchard grass, Figure 1), *Lolium perenne* L. (perennial ryegrass), *Poa pratensis* L. (Kentucky bluegrass), *Festuca arundinacea* L. (tall fescue), and *Phleum pratense* L. (Timothy grass). Seeds, obtained by the research unit of Agronomy and Crop Science (DSA3 Perugia), were planted in 9‐cm‐diameter pots on universal potting soil, and kept in growing chamber under 16 hr of light/8 hr of dark, 22°C. When grass blades reached about 15 cm in height, leaves were grinded in liquid nitrogen and total DNA was isolated. A small amount of pollen of the same species was collected directly from flowering plants in the urban area of Perugia.

For the constitution of the inclusivity and exclusivity panels of specificity, fresh leaves were collected from the Botanical Garden of Perugia, flour were bought from the supermarket, and certified reference materials for GMO (nonmodified DNAs and flour, American Oil Chemists’ Society—AOCS, USA. http://www.aocs.org/LabServices/crm) already present in the laboratory were used (Table 1).

**Table 1:** Inclusivity and exclusivity panels of specificity. Considered taxa, type of material from which DNA was obtained, and combinations of primers, tested in fast real‐time with matK‐PGP probe, are reported

System name						P1	P2	P3	P4	P5	P6	P7	P8	P9	P10
Primer forward						Ph matK 1‐F	Fe matK 2‐F	Poa matK 3‐F	Ph matK 3‐F	Da matK 4‐F	Ph matK 1‐F	Fe matK 2‐F	Poa matK 3‐F	Ph matK 3‐F	Poa matK 1‐F
Primer reverse						Poa matK 1‐R	Poa matK 1‐R	Poa matK 1‐R	Poa matK 1‐R	Poa matK 1‐R	Da matK 1‐R	Da matK 1‐R	Da matK 1‐R	Da matK 1‐R	Da matK 1‐R
	Family	Subfamily/Tribe	Genus	Species	Plant material										
Inclusivity panel															
	C+	mix Poideae				++	++	+++	++	+++	++	++	+++	++	++
**1**	Poaceae	Pooideae	* Poa *	* pratensis *	Leaf	++	**+**	+	+++	**++**	+		++	**++**	++
2	Poaceae	Pooideae	* Lolium *	* perenne *	Leaf	++	++	++	+	+++	**++**	+	++	+	+
3	Poaceae	Pooideae	* Dactylis *	* glomerata *	Leaf	++		++	++	+++	++	+	+++	+++	++
4	Poaceae	Pooideae	* Phleum *	* pratense *	Leaf	+++	+	++	++	++	++	+	++	++	+
5	Poaceae	Pooideae	* Festuca *	* arundinacea *	Leaf	++	+++	++	+	+++	+	++	++	+	+
6	Poaceae	Pooideae	*Triticum*	*durum*	Flour	++				++	++		+		
7	Poaceae	Pooideae	*Triticum*	*aestivum*	Flour	++		+		++	++		+		
8	Poaceae	Pooideae	*Triticum*	*dicoccum*	Flour	++				+++	++		+		
9	Poaceae	Pooideae	*Triticum*	*turgidum*	Flour	++		+		++	++		+		
10	Poaceae	Pooideae	*Secale*	*cereale*	Flour	++				++	++		+		
11	Poaceae	Pooideae	*Avena*	*fatua*	Leaf					+			+	+	
12	Poaceae	Pooideae	*Bromus*	*inermis*	Leaf	+		+		++	+		+		
13	Poaceae	Pooideae	*Hordeum*	*murinum*	Leaf	++		+		++	++		+		
16	Poaceae	Pooideae	*Hordeum*	*vulgare*	Flour	++				++	++		+		
14	Poaceae	Ehrhartoideae	*Oryza*	*sativa*	Leaf^a^			+							
15	Poaceae	Panicoideae	*Zea*	*mays*	Flour^b^	+		+			+				
17	Poaceae	Panicoideae	*Sorghum*	*vulgare*	Flour	+		++			+				
18	Poaceae	Panicoideae	*Panicum*	*miliacearum*	Flour	+		+			+				
19	Poaceae	Arundinoideae	*Phragmites*	*australis*	Leaf						+				
20	Poaceae	Danthonioideae	*Cortaderia*	*selloana*	Leaf										
21	Poaceae	Bambusoideae	unknown	unknown	Leaf	+		++			+		++		
Exclusivity panel															
1	Oleaceae	Oleoideae	*Olea*	*europaea*	Leaf	nt	nt	nt			nt	nt	nt	nt	nt
2	Urticaceae	Parietarieae	*Parietaria*	*officinalis*	Leaf	nt	nt	nt			nt	nt	nt	nt	nt
3	Fagaceae	Quercoideae	*Quercus*	*ilex*	Leaf	nt	nt	nt			nt	nt	nt	nt	nt
4	Cupressaceae	Cupressoideae	*Cupressus*	*sempervirens*	Leaf	nt	nt	nt		*	nt	nt	nt	nt	nt
5	Salicaceae	Saliceae	*Salix*	*babylonica*	Leaf	nt	nt	nt			nt	nt	nt	nt	nt
6	Salicaceae	Saliceae	*Populus*	*alba*	Leaf	nt	nt	nt			nt	nt	nt	nt	nt
7	Pinaceae	Pinoideae	*Pinus*	*pinea*	Leaf	nt	nt	nt			nt	nt	nt	nt	nt
8	Asteraceae	Asteroideae	*Artemisia*	*absithium*	Leaf	nt	nt	nt			nt	nt	nt	nt	nt
9	Solanaceae	Solanoideae	*Solanum*	*melongena*	Leaf	nt	nt	nt			nt	nt	nt	nt	nt
10	Solanaceae	Solanoideae	*Capsicum*	*annuum*	Leaf	nt	nt	nt			nt	nt	nt	nt	nt
11	Malvaceae	Malvoideae	*Gossypium*	*hirsutum*	Flour^c^	nt	nt	nt			nt	nt	nt	nt	nt
12	Brassicaceae	Brassiceae	*Brassica*	*napus*	Leaf	nt	nt	nt			nt	nt	nt	nt	nt
13	Fabaceae	Faboideae	*Lens*	*culinaris*	Leaf	nt	nt	nt			nt	nt	nt	nt	nt
14	Fabaceae	Faboideae	*Vicia*	*faba*	Leaf	nt	nt	nt			nt	nt	nt	nt	nt
15	Rosaceae	Rosoideae	*Rubus*	*idaeus*	Leaf	nt	nt	nt			nt	nt	nt	nt	nt
16	Betulaceae	Coryloideae	*Corylus*	*avellana*	Leaf	nt	nt	nt		*	nt	nt	nt	nt	nt
17	Asteraceae	Asteroideae	*Ambrosia*	*artemisiifolia*	Leaf	nt	nt	nt			nt	nt	nt	nt	nt
18	Apiaceae	Selinea	*Angelica*	*sylvestris*	Leaf	nt	nt	nt			nt	nt	nt	nt	nt

+++, *C*_q_ < 20; ++, 20 ≤ *C*_q_ < 30; +, *C*_q_ ≥ 30; empty box, negative result; *, unspecific amplification, with *C*_q_ > 36; nt, not tested.

a Nonmodified rice leaf tissue genomic DNA AOCS 0306‐D3.

b Nonmodified maize powder AOCS 0406‐A.

c Nonmodified cotton powder AOCS 0804‐A.

### 2.3 Preparation of aerobiological samples, DNA extraction, and quality/quantity assessment

A melinex section corresponding to 1 day of sampling (48 mm × 18 mm) had to be collected in a 2‐ml tube in order to perform DNA extraction. To make it possible, the daily sections were cut into smaller pieces following different strategies. Addition in the tube of five tungsten beads was also tested.

For gravimetric samples (Petri dishes), no preparation was needed: After exposure, they were simply plugged and sealed with parafilm until the beginning of the extraction protocol, when the first reagent was added directly in them.

DNA was extracted from leaves and flour by a cetyltrimethylammonium bromide (CTAB)‐based method, in accordance with the ISO 21571 2005/ADM 1:2013, and purified with NucleoSpin gDNA Clean‐up (Macherey‐Nagel).

DNA was isolated from loose Poaceae pollen grains and aerobiological samples on different supports (melinex tape or Petri dish) using a modified CTAB method (Torricelli et al., 2016). Sterile distilled water was used as negative control for DNA extraction in each session. The protocol was tested also in association with the purification kit NucleoSpin^**®**^ gDNA Clean‐up (Macherey‐Nagel). Real aerobiological samples collected in days with similar atmospheric pollen concentration were used as replicates in the melinex preparation and DNA extraction procedure tests. Four replicates for each combination listed in Table 2 were analyzed.

**Table 2:** Different types of sample preparation tested prior to the extraction of DNA from daily melinex sections

	Whole daily melinex/longitudinal half	Spiraled/Cut	Number of pieces	Tungsten beads added (Yes/No)	Ranking (based on average yield)
I	Whole	Cut	12	Yes	2
II	Whole	Cut	12	No	1
III	Half	Cut	6	Yes	6
IV	Half	Cut	6	No	4
V	Half	Spiraled^a^	–	Yes	5
VI	Half	Spiraled^a^	–	No	3

a Kraaijeveld et al. (2015).

For weekly melinex extraction, daily tubes were prepared (combination II, Table 2) and extracted with the same protocol, apart from the elution phase, when only the tube corresponding to the first day of the week was added with 50 μl of sterile distilled water and incubated in thermomixer at 56°C for 1 min. After that, the DNA solution was transferred in the second tube and incubated like the first. The steps were repeated for all the seven tubes, in order to bulk DNAs from particles captured during the whole week.

The concentration of the extracted nucleic acids was determined both by Qubit™ dsDNA BR Assay kit (Thermo Fisher Scientific) and fluorimeter (BioSpectrometer^®^ fluorescence, Eppendorf) for DNA from *D. glomerata, L. perenne, F. arundinacea, P. pratense* and *P. pratensis* leaves and loose pollen. For samples which were under the detection level of the instruments, or did not need precise quantification, estimates of quantity and quality of the extracted DNA were assessed by inhibition test (Waiblinger & Grohmann, 2014) through fast real‐time PCR, using as reference genes *actin* and/or *tRNA‐Leu* (Laube et al., 2010; primer–probe sets are shown in Table 3). The test consisted in analyzing two fast real‐time PCR replicates of undiluted DNA and its dilution 1:4 for each extracted sample. In absence of inhibitors, the difference between the measured mean cycle threshold (*C*_q_) of undiluted and diluted DNA (Δ*C*_q_) should be of 2, with an acceptability range of 1.5—2.5.

**Table 3:** List of the all oligonucleotides used in this work

Name	Sequence (5′–3′)	Amplicon length (bp)	Reference
act‐f^a^	CAAGCAGCATGAAGATCAAGGT	~103	Laube et al. (2010)
act‐r^a^	CACATCTGTTGGAAAGTGCTGAG		
act probe^a^	FAM—CCTCCAATCCAGACACTGTACTTYCTCTC—TAMRA		
tRNALeu‐f^a^	ATTGAGCCTTGGTATGGAAACCT	~90	
tRNALeu‐r^a^	GGATTTGGCTCAGGATTGCC		
tRNALeu‐probe^a^	FAM—TTAATTCCAGGGTTTCTCTGAATTTGAAAGTT—TAMRA		
F‐for (*waxy*)^b^	TGCGAGCTCGACAACATCATGCG	~350	Mason‐Gamer et al. (1998)
K‐bac (*waxy*)^b^	GCAGGGCTCGAAGCGGCTGG		
rbcL‐F^b^	TTGCAAAGGTTTCATTTACGC	~750	Drumwright et al. (2011)
rbcL‐R^b^	TACCTGCAGTCGCATTCAAG		
matK 390‐F^b^^,^ c	CGATCTATTCATTCAATATTTC	~850	Cuénoud et al. (2002)
matK 1326‐R^b^^,^ c	TCTAGCACACGAAAGTCGAAGT		
Da matK 1‐R	CGATCAAGAATATCCCAATCTGAC	Various combinations and lengths	This work
Da matK 1‐F	TCTTGCTTTGATTTTATGGGGT		
Da matK 4‐F	ATACCATAGTTCCCGCTACTGT		
Ph matK 1‐R	ATCCGACCAAATCGATCAAG		
Ph matK 1‐F	GAATCAAATGCTGGAGAATTCG		
Ph matK 3‐R	TGCATTCGAGTATCTATTAGAAAC		
Ph matK 3‐F	GTACCTTATCCATTTGTGGC		
Lo matK 1‐R	GGTACCCCATAAAATCAAAGCA		
Lo matK 1‐F	CTTTTTGCATCAAAAGGTACTCC		
Fe matK 2‐F	CCAAAAAGTCCTTTCTTAGTAAAGAATA		
Poa matK 1‐R	TTTCTACATATCCGACCAAACC		
Poa matK 1‐F	CATTTCTAATAGATACTCGAATGCC		
Poa matK 3‐R	GGGAACTATGGTATCGAATTTTG		
Poa matK 3‐F	CGCGAAGGATCCATCTAAACC		
matK‐PGP Probe	FAM—GGATACTTATCAAAAGCTCA—MGB		

a Used to detect reference genes in DNA quality/quantity assessments.

b Used for a first sequence comparison between the five species of interest.

c Used to generate matK sequences on which all the other primers and the probe have been designed.

Plant DNAs used in specificity test were checked with *actin* fast real‐time PCR inhibition assay, and concentrations were adjusted based on a *C*_q_ of 29‐30 when necessary (Torricelli et al., 2016).

In fast real‐time assay results, *C*_q_s = undetermined are negative results, for no amplification observed.

### 2.4 Primers and probe design

Three pairs of primers, directed to different parts of the plant genome, were found in bibliography (Cuénoud et al., 2002; Drumwright, Allen, Huff, Ritchey, & Cahoon, 2011; Mason‐Gamer, Weil, & Kellogg, 1998) and used to perform a first screening of the five species of interest. They were directed to the nuclear *waxy* gene (F‐for/K‐bac), and the plastidial *rbcL* (rbcl‐F/rbcl‐R) and *matK* (matK 390‐F/matK 1326‐R) genes, which have been often targeted in barcoding approaches (Table 3). Primers (Sigma‐Aldrich) were used to amplify leaf DNA under the published conditions. All the PCR products were purified using the illustra GFX PCR DNA and GEL Band Purification kit (GE Healthcare), and Sanger sequenced. Sequences were aligned using MEGA6 Software (Tamura, Stecher, Peterson, Filipski, & Kumar, 2013). *matK* sequences, of higher quality, were analyzed to identify polymorphic regions. New primer pairs with different levels of discriminating power (subfamily/species) were designed using Primer3 (Untergrasser et al., 2012). The matk‐PGP TaqMan probe, for real‐time PCR assay, was designed using Primer Express software 3.01 (Thermo Fisher Scientific), to hybridize a conserved trait of *matK* sequences from the five studied species. A 6‐carboxyfluorescein (FAM) dye was linked at the 5′‐end and a non–fluorescent minor groove binder quencher (MGB) at the 3′‐end of the probe. Probe was purchased from Thermo Fisher Scientific. The oligonucleotide sequences are listed in Table 3. The alignment of Poaceae *matK* fragment on which the sequences were studied is shown in Figure S1.

### 2.5 Fast real‐time PCR

Real‐time PCRs were run in a 7900HT Fast Real‐Time PCR System (Thermo Fisher Scientific), in a reaction volume of 20 μl. Those with TaqMan chemistry (used for specificity/sensitivity tests and for the screening of real samples with subfamily‐specific primers) were performed in fast mode, with 1× of TaqMan^®^ Fast Universal PCR Master Mix (2×) No AmpErase UNG (Thermo Fisher Scientific), 900 nmol/L of each primer, 200 nmol/L of probe, 2 μl DNA, using the following cycling conditions: 95°C for 20 s, 40 cycles with 95°C for 3 s, and 60°C for 30 s. The annealing temperatures of 65, 66, and 67°C were also tested for the species‐specific detection of *D. glomerata, P. pratense, F. arundinacea*, and *P. pratensis*.

In the nested fast real‐time PCR, 40 initial rounds of amplifications were performed as indicated in TaqMan chemistry, with Ph matK 3‐F and Poa matK 1‐R primers (P4 system, see further), but without probe. For the second step, 1 μl of the PCR product from the initial amplification was used as template in a subsequent fast real‐time PCR of TaqMan chemistry, performed in the same conditions described above (other 40 cycles) with matK‐PGP probe‐ and species‐specific primer couples at appropriate annealing temperatures. Control reactions in the absence of template (NTC, no‐template control) were included in each assay.

A SYBR Green assay (Thermo Fisher Scientific) was also tested for the assessment of primer specificity, but it did not allow discrimination and thus was not used eventually.

### 2.6 Specie‐specific and subfamily (Pooideae)‐specific methods

The alignment and polymorphism analysis of *matK* amplicons allowed the design of several primers, which were combined and tested in order to find the right pair for each species. Suitable couples were tested with SYBR Green and TaqMan (with matk‐PGP probe) chemistries on leaf DNA from all the studied species. For primer combinations deemed appropriate, stringency was heightened gradually, increasing annealing temperatures until only the right species amplified.

Given the high number of available primer combinations, the possibility of other identification approaches was tested. Ten studied couplings of primers (systems P1–P10) were used to screen, by TaqMan matk‐PGP real‐time PCR, an inclusivity panel composed of DNAs from Poaceae species (including the five studied) belonging to several subfamilies. Those which gave interesting amplification profiles were tested also versus an exclusivity panel of plants belonging to other families to verify specificity; discrepant results were verified exploiting published *matK* sequences and BLAST (https://www.ncbi.nlm.nih.gov). Eventually, P4 and P5 systems were selected to be used in the analysis of real aerobiological samples. Primer combination for each system, and inclusivity and exclusivity panel species are listed in Table 1.

To test the sensitivity of developed systems, fast real‐time PCRs were performed on scalar dilutions of the template (leaf DNA of correspondent species for the species‐specific ones, and a homogeneous mix of leaf DNA from the five grasses for P5), in order to establish the minimum quantity of target that should be present in a sample to be detected.

Systems were finally used to screen a selection of DNAs among those extracted from real aerobiological samples. In this phase, a mix of DNA from *D. glomerata, L. perenne, P. pratense, F. arundinacea,* and *P. pratensis* leaves, at a concentration of 30 ng/μl, was used as positive control for the Pooideae‐specific method. Two mixtures of DNA were used as positive direct templates in the second step of the nested approach (the species‐specific one): One was the same as above, but at a concentration of 15 ng/μl, while the second was a homogeneous mix of pollen DNA from the same species.

## 3 RESULTS

### 3.1 Aerobiological data

**Figure 2:**
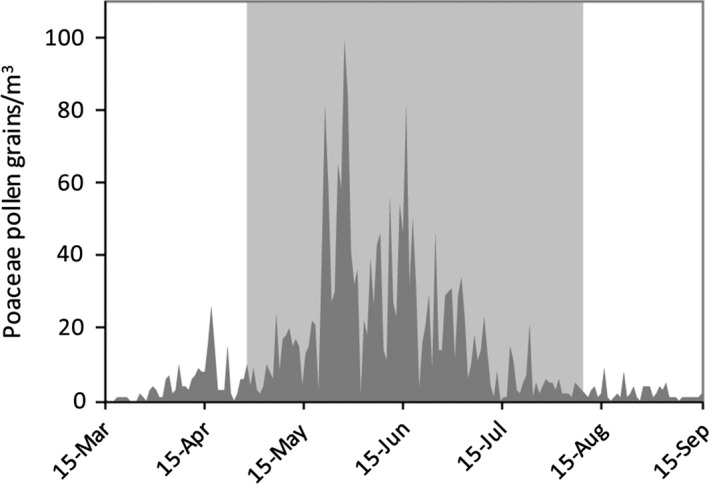
Trend of atmospheric grass pollen concentration in the area of the study, 2016. The period of maximum pollen emission is evidenced

In 2016, the grass pollen season in Perugia (Central Italy) started on May 1 and ended on August 12. The API (cumulative sum of the daily concentrations registered along the season) was 2,443, and the peak was reached on May 28, with a value of 99 Poaceae pollen grains/m^3^. Figure 2 reports the trend of daily atmospheric concentration values.

### 3.2 Comparison between different DNA extraction strategies

The first trial consisted in applying the existing extraction protocol on some randomly selected daily melinex samples (corresponding to April 30, May 7, and May 9, prepared like in combination II, Table 2) and 30 mg of loose *D. glomerata* pollen. At the end of the protocol, the presence and quality of total nucleic acids was evaluated trough the inhibition test with fast real‐time PCR, using *actin* as reference gene (theoretically present in single copy in all eukaryotic cells), before and after the treatment with the purification kit. Double elution was also tested for pollen. *C*_q_s values showed that the protocol is suitable for DNA extraction from the tested matrixes, but the purification after extraction is not convenient as it reduces nucleic acid yield (Table S1).

**Figure 3:**
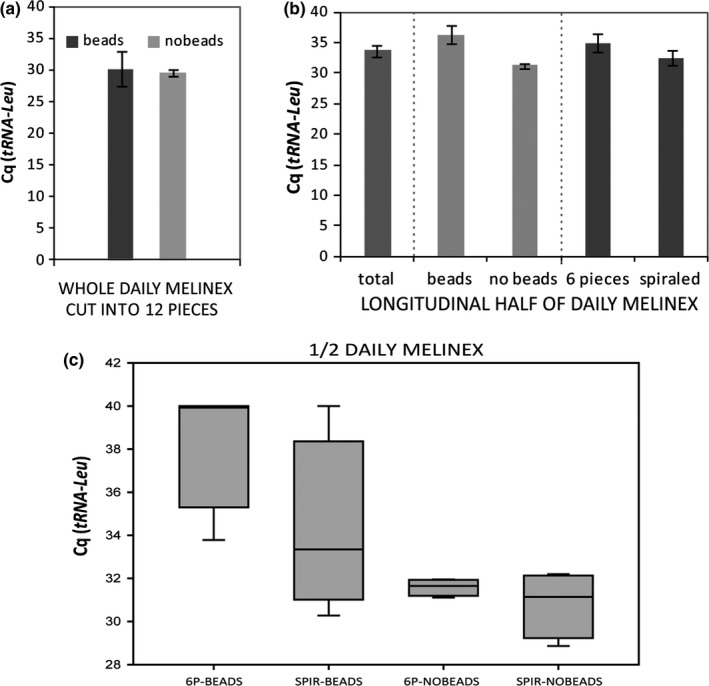
Evaluation of different sample preparation strategies prior to extraction. Lower *C*_q_ corresponds to faster achievement of the threshold, indicating a higher presence of template in the sample and, thus, a more efficient extraction. (a) When using the whole daily melinex, adding tungsten beads does not lead to a significant increase in yield. (b) In the treatment of half daily melinex, yields are better than the average (total) in the absence of beads, while cutting or spiraling the tape does not lead to significant differences. (c) Boxplot showing results obtained with the four different combinations of sample preparation methods

Daily melinex tapes collected in days in which similar atmospheric pollen concentrations (between 100 and 200 pollen grains/ m^3^ of air) were registered were chosen to compare the preparation methods listed in Table 2. In this case, after extraction, quantity and quality of DNA were assessed by means of fast real‐time PCR with *tRNA‐Leu* as reference gene (plastidial gene, present only in plant cells). One‐way ANOVA was applied on *C*_q_s obtained from samples cut into 12 pieces, treated or not with tungsten beads. A default *C*_q_ value of 40 was assigned to samples that did not show amplification. Statistics indicated that no significant difference exists between the two methods’ efficiency (Figure 3a). For daily melinex longitudinal halves, two‐way ANOVA showed no significant change in yield if spiraling the tape rather than cutting it into six pieces. The presence of beads, instead, seems to significantly reduce the quantity and quality of extracted nucleic acids (Figure 3b,c). No significant interaction was recorded between the cutting strategy and the presence/absence of beads. The average *C*_q_ of all the samples prepared with combination I and II is 29.46 ± 1.49, while with combinations III, IV, V, and VI it is 31.79 ± 1.50, confirming what is deductible, that is, using the whole daily melinex ensures greater DNA yield.

Some weekly samples (melinex tape and Petri dishes) were selected to evaluate DNA extraction from deposited pollen: *C*_q_s to *tRNA‐Leu* were analyzed, and results are reported in Table 4.

**Table 4:** Results of DNA extraction from weekly supports. *C*_q_s to *tRNA‐Leu*, as for daily samples, reflect the total plant DNA extracted

Sample name	Week	Cumulative pollen concentration (grains/m^3^)	Cumulative Poaceae pollen concentration (grains/m^3^)	Support	Mean *C*_q_ (*tRNA‐Leu*)
1824M	April 18–24	1,540	41	Melinex	25.16
305M	May 30–June 5	1,889	189	Melinex	24.52
305P				Petri dish	26.71
612M	June 6–12	1,596	223	Melinex	25.71
612P				Petri dish	27.91
410P	July 4–10^a^	537	105	Petri dish	Und
1824P	July 18–24	303	45	Petri dish	30.40
814P	August 8–14	411	18	Petri dish	28.42
2228P	August 22–28^a^	305	20	Petri dish	34.73^b^
294P	August 29–September 4^a^	306	23	Petri dish	Und

a Technical problems during extraction.

b Δ*C*_q_ > 2.5, low‐quality DNA.

### 3.3 Development and optimization of species‐specific primers

The primers, designed on single nucleotide polymorphisms (SNPs) between *matK* sequences of the species of interest, were coupled in several combination in order to obtain species‐specific amplifications. The selected couples were tested on the same amount of leaf DNA for each grass. SYBR Green chemistry did not allow a discrimination, due to the nonspecific and not‐saturating nature of the nucleic acid‐binding dye (Table S2). In TaqMan matK‐PGP systems, the annealing temperature (*T*_a_) of 60°C was firstly applied, and couples that gave sensibly lower *C*_q_s for the target species were tested at higher stringency condition, raising the *T*_a_ until a single species gave amplification (Table S3). Eventually, couples of species‐specific primers were obtained for *D. glomerata*,* P. pratense*,* F. arundinacea,* and *P. pratensis* (Table 5). No identification system was found for *L. perenne*, as its specific SNPs were too far from *matK*‐PGP probe position in the sequence. For the four species‐specific systems, sensitivity was also assessed (Table S4) and final results are reported in Table 5.

**Table 5:** Species‐specific systems

TaqMan^®^ matK‐PGP probe				
	*D. glomerata*	*P. pratense*	*F. arundinacea*	*P. pratensis*
System Name	Da 2	Ph 1	Fe 1	Poa 1
Primer for	Da matK 4‐F	Ph matK 1‐F	Fe matK 2‐F	Poa matK 1‐F
Primer rev	Da matK 1‐R	Ph matK 1‐R	Ph matK 1‐R	Poa matK 1‐R
*T*_a_	67°C		66°C	
Sensitivity (ng)	0.15	0.15	0.6	6

*Dactylis glomerata*,* D. glomerata*;* Phleum pratense*,* P*. *pratense*;* Festuca arundinacea*,* F*. *arundinacea*;* Poa pratensis*,* P*. *pratensis*.

### 3.4 Development and optimization of subfamily‐specific methods

Aligning the sequences of the five species of interest with *matK* sequences from other Poaceae found in bibliography, the possibility to obtain systems with different levels of specificity emerged. New combinations of the designed primers were identified (all suitable for the use with TaqMan matK‐PGP probe) and tested on an inclusivity panel of grass species belonging to several subfamilies (Table 1). P5 showed an interesting positivity profile, including only specimens from Pooideae subfamily. P4 and P10 also caught the attention, amplifying exclusively the five species object of this study. Moreover, P4 amplicon includes those from all four developed species‐specific systems, and thus was chosen to be used as pre‐amplification system in a nested PCR approach with them. P4 and P5 were tested also versus an exclusivity panel of species belonging to other subfamilies. Only two cases of slight unspecific amplification were highlighted: cypress (*Cupressus sempervirens*) and hazel (*Corylus avellana*) (Table 1). *matK* sequences of these species, found in databases, were blasted versus grass *matK* sequences. No significant similarity was found for the former, while for the latter, the alignment revealed a 76% of sequence identity in the DNA region comprised between the primers of P5. The sensitivity of this system was also assessed, and its limit of detection resulted to be of 0.0002 ng of template.

### 3.5 Screening of real samples

The developed identification systems (species‐ and subfamily‐specific) were finally tested on a set of DNAs extracted from real aerobiological samples. First trials with systems listed in Table 5 on real samples did not allow the detection of the species, probably due to extremely low levels of template. Most likely for the same reason, P4 system alone did not gave detectable amplification (data not shown). But when they were coupled in the nested PCR to increase the concentration of target DNA, amplification appeared. Fast real‐time with P5 primers revealed the presence/absence of pollen from grasses belonging to Pooideae subfamily. Reference days of the analyzed samples, with pollen concentrations registered through classic aerobiological monitoring, and fast real‐time outcomes of the applied systems, are presented in Table 6.

**Table 6:** List of the analyzed aerobiological samples, with the DNA extraction method applied (numbers referring to Table 2) and results of fast real‐time PCR with different identification systems (samples which did not amplify *tRNA‐Leu* were not further analyzed and are not shown). For the species‐specific systems, only the positivity (+) is reported

Reference period (2016)	Aerobiological information		Sample	Extraction method	*tRNA‐ Leu* (*C*_q_)	P5 (*C*_q_)	Nested with P4			
	Pollen/m^3^	Poaceae pollen/m^3^					Da 2	Ph 2	Fe 1	Poa 1
26‐April	149	6	26A‐6P.	3	33.78	Und				
			26A‐S.	5	33.23	Und				
05‐May	140	8	5M‐6P	4	31.11	37.62		+	+	+
			5M‐S	6	31.94	Und				
06‐May	166	6	6M‐12P	2	25.71	35.27	+	+	+	+
08‐May	108	8	8M‐6P	4	31.87^a^	39.16	+	+		
			8M‐S	6	30.34	39.81				
10‐May	129	18	10M‐S.	5	30.28	38.96	+	+	+	+
13‐June	125	23	13G‐12P	2	27.33	34.67				
14‐June	193	54	14G‐12P	2	31.42	35.17		+		
17‐June	187	31	17G‐12P.	1	29.71	Und				
18‐June	162	50	18G‐12P.	1	29.55	39.32				
21‐June	131	16	21G‐6P.	3	39.72^a^	Und				
			21G‐S.	5	33.45	Und	+			
23‐June	127	29	23G‐12P.	1	32.82^a^	Und				
25‐June	184	46	25G‐S	6	30.56	36.12				
			25G‐S.	5	30.91	Und				
28‐June	115	29	28G‐6P	4	31.43	37.43		+		
			28G‐S	6	32.20	37.48	+	+	+	
29‐June	173	30	29G‐S	6	32.22	37.68				
			29G‐S.	5	30.62	38.57				
30‐June	164	31	30G‐S	6	32.56	36.03	+	+	+	
			30G‐S.	5	30.90	38.15				
02‐July	125	29	2L‐6P	4	31.95	38.10		+	+	
			2L‐S	6	28.86	Und				
03‐July	169	34	3L‐S	6	31.14	35.91			+	
			3L‐S.	5	28.46	Und				
04‐July	117	23	4L‐12P	2	29.10	38.39	+			
07‐July	122	5	7A‐12P	2	30.04	38.34	+			

	∑Pollen/m^3^	∑Poaceae pollen/m^3^		Weekly						
18–24 April	1,540	41	1824P	Melinex	25.16	34.87	+		+	
30 May–5 June	1,889	189	305M	Melinex	24.52	29.08	+	+	+	+
			305P	Petri dish	26.71	33.6	+	+	+	+
6–12 June	1,596	223	612M	Melinex	25.71	30.49	+	+	+	+
			612P	Petri dish	27.91	34.33	+	+	+	+
18–24 July	303	45	1824P	Petri dish	30.40	38.77				
8–14 August	411	18	814P	Petri dish	28.42	Und				
22–28 August	305	20	2228P	Petri dish	34.73^a^	Und				

a Δ*C*_q_ <1.5 or >2.5, low‐quality DNA.

## 4 DISCUSSION

The application of the pollen DNA extraction protocol, developed by Torricelli et al. (2016), allowed to isolate genetic material of generally good quality from aerobiological samples collected by traps and loose *D. glomerata* pollen grains. Nevertheless, nucleic acid concentration was below the instrumental detection level, and an evaluation of quantity was made through fast real‐time PCR toward *actin* gene (Table S1). *C*_q_s were high on average—close to the threshold value of 40—reflecting a low concentration of template DNA (that could be due to a low number of starting biological particles and/or low efficiency of the method), but quality was good (Δ*C*_q_s comprised between 1.5 and 2.5) in all the extractions, except one. Both quantity and quality lowered after treatment with the purification kit, and therefore, this step was eliminated in all subsequent extractions. When *actin* (which is present in all eukaryotic cells) was used as reference gene, not only DNA from pollen, but also from fungi and small insects was probably amplified. However, being it a nuclear, single‐copy gene, its presence in such samples is of difficult detection. For this reason, we decided to skip to *tRNA‐Leu* as reference gene in the following analysis. The chosen primer–probe system, designed by Laube et al. (2010), is plant specific, allowing the detection of DNA extracted from pollen only. Moreover, it is plastidial, and therefore present in multiple copy in each cell, pollen cells comprised (Tang et al., 2009), raising the amplification signal in fast real‐time PCR. On the other hand, referring to a multiple‐copy gene has the disadvantage to impede precise quantitative evaluations, because every species, and potentially every pollen grain, carry a different number of plastids. Another way to evaluate the relative efficiency of the different extraction procedures would have been the constitution of realistic artificial samples, containing pollen grains from a legit mix of *taxa*, in a total fixed amount for each type, resembling the average quantity of grains that can be found on a spring–summer aerobiological daily slide. Unfortunately, pure loose pollen from different plants was not available in the context of this study. The alternative strategy proposed was to use real aerobiological samples collected in days with similar atmospheric pollen concentration as replicates. We selected melinex fragments collected during 2016 Poaceae pollen season, corresponding to days in which an atmospheric concentration of total pollen between 100 and 200 grains/m^3^ was registered. This value was chosen being the most frequent along the sampling period, and reflecting a not “extreme” aerobiological situation (too low or too high pollen presence in the atmosphere). Poaceae pollen concentration of the same days oscillated between 5 and 54 grains/m^3^, comprising values definable low (1–25 grains/m^3^), medium (26–50 grains/m^3^), and high (above 50 grains/m^3^) (Galán, Cariñanos, Alcázar, & Domínguez, 2007). *C*_q_s of the extracts to *tRNA‐Leu* were analyzed by ANOVA to understand which sample preparation method leads to better DNA yields. Not surprisingly, the use of the whole daily melinex section resulted preferable. When extracting from half sample, spiraling the melinex or cutting it into six pieces did not lead to significantly increased efficiency (Figure 3), but the former procedure, proposed by Kraaijeveld et al. (2015), seemed practically easier, faster, and less “disturbing” for the sample surface. The addition of beads resulted not significantly favorable when using the whole tape section, and reduced the efficiency in half melinex samples (Figure 3): This is probably due to excessive beating, which can cause nucleic acid shredding. Overall, this passage resulted not convenient in our study, in terms of yield, practicality, and costs, although bead beating is provided in other published protocols for DNA extraction from aerobiological matrixes (Kraaijeveld et al., 2015; Mohanty et al., 2017). Melinex fragments were observed under the microscope after DNA extraction treatments: Independently from the preparation method, the almost totality of the pollen grains were removed from the surface. Extraction from weekly melinex is convenient as it bulks DNA from 7 days, increasing the amount of template available for subsequent analysis. When comparing its *C*_q_s with those from Petri dishes (Table 4, weeks May 30–June 5 and June 6–12), weekly melinex gave higher yields. Nonetheless, with the exception of a couple of samples which underwent technical problems during extraction, DNA recovery from dishes was possible with the proposed protocol (Table 4).

The second phase of this research regarded the assessment of molecular methods capable to identify grass pollen from aerobiological samples at a higher level than the family. Metabarcoding approaches, based on high‐throughput sequencing, have already been proposed for the analysis of pollen mixed samples (Keller et al., 2015; Kraaijeveld et al., 2015; Sickel et al., 2015), but they require expensive machinery and trained bioinformaticians capable to manage the huge data outcome. Mohanty et al. (2017), instead, used qPCR to analyze aerobiological samples quickly, verifying the presence and quantity of *Juniperus* species. To our knowledge, no information has been published about molecular analysis of airborne grass pollen to integrate classic aerobiological data.

Here, five among the most widespread and allergenic Poaceae species in Europe were selected as target for a barcoding approach. When aligning their *matK* sequences, high similarity was observed (Figure S1), but several SNPs could be exploited to draw species‐specific primer. The first identification attempt, with SYBR Green chemistry, did not allow a discrimination (Table S2), and thus, we decided to switch to TaqMan^®^, designing a general probe to be coupled with species‐specific primers. Unfortunately, this change of strategy led to the impossibility to develop primers for *Lolium perenne*, but it was still used as control (alone or in the Poaceae mix samples) in following trials and in subfamily‐specific systems. Species‐specific systems were obtained for the four remaining species (Table 5), even if their sensitivity levels are not excellent (Table S4), probably due to the highly stringent PCR conditions needed to avoid nonspecific pairing of the primers (Table S3). When observing the amplification profile of Pooideae methods (P1 to P10, Table 1), P4 showed as a good tool to pre‐amplify the evidently low quantity of DNA from the five species of interest present in the samples, and perform a nested PCR with species‐specific systems. All the samples that had resulted positive to *tRNA‐Leu* were screened with the nested approach. Presence/absence of the species was evaluated (Table 6—only qualitatively), but the pattern of appearance along the grass pollen season was not always clear. For example, it is not likely to find flowering *P. pratense* at the beginning of May, or *F. arundinacea* and *D. glomerata* at the end beginning of July, in Central Italy (Frenguelli et al., 2010; Ghitarrini, Tedeschini, et al., 2017), even if phenomena of transport from other latitudes and/or resuspension after deposition could be involved. Sometimes species were detected in a half of the sample and not in the other (e.g., May 8, June 30), and this can indicate low efficiency of extraction and/or of detection. The outcome seemed much more reliable when screening weekly samples, both from melinex and Petri dishes. The period of main pollen emission for Poaceae, in the area of the study (2016), started on May 1. In fact, in week 18 April 2016 to 24 April 2016, only early flowering species (*D. glomerata* and *F. arundinacea*) were detected. On the other hand, all the four species were present consistently in samples collected between May 30 and June 12, coinciding with the period of maximum pollen emission, just after the peak, registered on May 28. Late season samples (July 18–24, August 8–14, August 22–28) showed a legit absence of the species, as in the considered area, this period is commonly dominated by non‐Pooideae grasses, such as *Cynodon*,* Digitaria*,* Setaria,* and *Echinocloa* species (Ghitarrini, Tedeschini, et al., 2017). Overall results suggest that, until the DNA extraction protocol for aerobiological daily mixed samples is not optimized, the extraction on weekly basis ensures a higher amount and better representativeness of the templates for qualitative identification at species level.

The subfamily‐specific system, P5, showed a good level of sensitivity, compared to those of the species‐specific primers. The unspecific amplification signals showed in the test toward the exclusivity panel (with *C. sempervirens* and *C. avellana*), besides being very weak, are not considered threatening for its informative power. In fact, in the case of cypress, no significant similarity of sequence has been evidenced in the blast analysis, indicating a probable contamination of the plant material. In the case of hazel instead, *matK* sequence has a certain level of similarity with Poaceae, but their pollination periods are not overlapping (Italian Association of Aerobiology—http://www.ilpolline.it). Thus, a false‐positive result due to the presence of this *taxon* in aerobiological samples collected during grass pollen season is unlikely. The screening of real samples with P5 showed consistency with the species‐specific analysis: Samples that were positive to at least one of the four species were positive also to the subfamily system. Samples which were positive to P5 but negative to the species‐specific systems have to be interpreted like containing DNA from Pooideae, but not from *D. glomerata*,* P. pratense*,* F. arundinacea,* or *P. pratensis*. The only exception regarded sample 21G‐S., of June 21, which resulted negative to P5 but positive to Da 1. Here, probably, the amount of cumulative DNA from Pooideae in the original sample did not reach the limit of detection of P5 (0.0002 ng), but after nested PCR with P4, DNA from *D. glomerata* was enough to be positive to Da 1.

In light of the observations made in this study, an efficient extraction of nucleic acids from aerobiological matrixes is still a critical point for their biomolecular analysis. Pollen grains from different taxa have different physical features, and the treatment that is sufficient for breaking one type of pollen wall could not be enough for another. In order to obtain reliable information from the analysis of airborne pollen DNA, extraction protocols have to be optimized, ensuring the recovery of genetic material from all the pollen *taxa* present in a sample. Moreover, to improve the efficacy in identification, *matK* and other sequences could be further dissected; high‐throughput sequencing approaches could allow the identification of a wider SNP pattern in the species of interest, and species‐specific probes could be designed on the polymorphisms.

## 5 CONCLUSIONS

The objective of this study was to explore the possibility of developing a fast and easy protocol to obtain specific information about grass pollen presence in the atmosphere, through molecular techniques. Several methods were presented to collect pollen grains with the purpose of extracting their DNA, even if the absolute efficiency of these procedures could not be completely assessed. Generally, the weekly methods seem to lead to more representative results. Important polymorphisms were identified in species of interest, and species‐specific primers were designed to be used in TaqMan fast real‐time PCR, with a “universal” probe. This system is more sensible than a classic endpoint PCR, but cheaper than an allelic discrimination. Exploiting the same probe, also a Pooideae‐specific system (P5) was developed, which could be a useful tool to highlight the presence of the most allergenic grasses in aerobiological samples. Difficulties arose from the low quantity and high complexity of the biologic material in the starting sample; in fact, recovery and identification of nucleic acids becomes more and more an issue, as the target becomes narrower.

Overall, the basis was laid for a fast molecular technique to be used in the dissection of grass pollen season, to obtain data about the species present in the atmosphere in a certain moment. Information obtained with classic aerobiological monitoring was enriched, even if further investigation is needed to make the whole methodology more reliable, and bring it to a quantitative level. However, results presented in this work are a good starting point for studies aimed at the molecular identification of airborne Poaceae pollen, which would be an important support for next‐generation allergological medicine.

## CONFLICT OF INTEREST

None declared.

## DATA ACCESSIBILITY

All relevant data are within the paper and its Supporting Information files.

## AUTHOR CONTRIBUTIONS

E. A. and G. F. conceived the idea for this study and critically revised the manuscript. S. G. and E. P. designed the research, performed the analysis, and drafted the manuscript. C. R. provided ideas to improve DNA extraction from pollen. E. T. contributed to the collection of aerobiological samples. G. R. T. helped performing real‐time PCR assays. All the authors read and approved the final version of the manuscript.

## References

[ece33891-bib-0001] Andersson, K. , & Lidholm, J. (2003). Characteristics and immunobiology of grass pollen allergens. International Archives of Allergy and Immunology, 130(2), 87–107. https://doi.org/10.1159/000069013 12673063 10.1159/000069013

[ece33891-bib-0002] Annesi‐Maesano, I. , Rouve, S. , Desqueyroux, H. , Jankovski, R. , Klossek, J. M. , Thibaudon, M. , … Didier, A. (2012). Grass pollen counts, air pollution levels and allergic rhinitis severity. International Archives of Allergy and Immunology, 158(4), 397–404. https://doi.org/10.1159/000332964 22487690 10.1159/000332964

[ece33891-bib-0003] Ball, T. , Sperr, W. R. , Valent, P. , Lidholm, J. , Spitzauer, S. , Ebner, C. , … Valenta, R. (1999). Induction of antibody responses to new B cell epitopes indicates vaccination character of allergen immunotherapy. European Journal of Immunology, 29(6), 2026–36. https://doi.org/10.1002/(sici)1521-4141(199906)29:06<2026::aid-immu2026>3.0.co;2-2 10382766 10.1002/(SICI)1521-4141(199906)29:06<2026::AID-IMMU2026>3.0.CO;2-2

[ece33891-bib-0004] Bell, K. L. , de Vere, N. , Keller, A. , Richardson, R. T. , Gous, A. , Burgess, K. , & Brosi, B. J. (2016). Pollen DNA barcoding: Current applications and future prospects. Genome, 59, 629–640. https://doi.org/10.1139/gen-2015-0200 27322652 10.1139/gen-2015-0200

[ece33891-bib-0005] Bosque, J. , Van Cauwenberge, P. , Khaltaev, N. , & in collaboration with World Health Organization (2001). Allergic rhinitis and its impact on asthma. Journal of Allergy and Clinical Immunology, 108(5), S147–S334. https://doi.org/10.1067/mai.2001.118891 11707753 10.1067/mai.2001.118891

[ece33891-bib-0006] Bousquet, J. , Anto, J. , Auffray, C. , Akdis, M. , Cambon‐Thomsen, A. , Keil, T. , … Zuberbier, T. (2011). MeDALL (Mechanisms of the Development of ALLergy): An integrated approach from phenotypes to systems medicine. Allergy, 66(5), 596–604. https://doi.org/10.1111/j.1398-9995.2010.02534.x 21261657 10.1111/j.1398-9995.2010.02534.x

[ece33891-bib-0007] Bruni, I. , Galimberti, A. , Caridi, L. , Scaccabarozzi, D. , De Mattia, F. , Casiraghi, M. , & Labra, M. (2015). A DNA barcoding approach to identify plant species in multiflower honey. Food Chemistry, 170, 308–315. https://doi.org/10.1016/j.foodchem.2014.08.060 25306350 10.1016/j.foodchem.2014.08.060

[ece33891-bib-0008] Burr, M. L. (1999). Grass pollen: Trends and predictions. Clinical & Experimental Allergy, 29(6), 735–738 https://doi.org/10.1046/j.1365-2222.1999.00621.x 10336586 10.1046/j.1365-2222.1999.00621.x

[ece33891-bib-0009] Chapman, M. D. (1998). Environmental allergen monitoring and control. Allergy, 53(45 Suppl.), 48–53. https://doi.org/10.1111/j.1398-9995.1998.tb04939.x 10.1111/j.1398-9995.1998.tb04939.x 9788707

[ece33891-bib-0010] Christenhusz, M. J. M. , & Byng, J. M. (2016). The number of known plants species in the world and its annual increase. Phytotaxa, 261(3), 201–217. https://doi.org/10.11646/phytotaxa.261.3.1

[ece33891-bib-0011] Cuénoud, P. , Savolainen, V. , Chatrou, L. W. , Powell, M. , Grayer, R. J. , & Chase, M. W. (2002). Molecular phylogenetics of Caryophyllales based on nuclear 18S rDNA and plastid *rbcL*,* atpB*, and *matK* DNA sequences. American Journal of Botany, 89(1), 132–144. https://doi.org/10.3732/ajb.89.1.132 21669721 10.3732/ajb.89.1.132

[ece33891-bib-0012] D'Amato, G. (2000). Urban air pollution and plant derived respiratory allergy. Clinical & Experimental Allergy, 30(5), 628–636. https://doi.org/10.1046/j.1365-2222.2000.00798.x 10792353 10.1046/j.1365-2222.2000.00798.x

[ece33891-bib-0013] D'Amato, G. , Cecchi, L. , Bonini, S. , Nunes, C. , Annesi‐Maesano, I. , Behrendt, H. , … van Cauwenberge, P. (2007). Allergenic pollen and pollen allergy in Europe. Allergy, 62(9), 976–990. https://doi.org/10.1111/j.1398-9995.2007.01393.x 17521313 10.1111/j.1398-9995.2007.01393.x

[ece33891-bib-0014] De Weger, L. A. , Beerthuizen, T. , Gast‐Strookman, J. M. , van der Plas, D. T. , Terreehorst, I. , Hiemstra, P. S. , & Sont, J. K. (2011). Difference in symptom severity between early and late grass pollen season in patients with seasonal allergic rhinitis. Clinical and Translational Allergy, 1(1), 18 https://doi.org/10.1186/2045-7022-1-18 22410160 10.1186/2045-7022-1-18 PMC3339365

[ece33891-bib-0015] Drumwright, A. M. , Allen, B. W. , Huff, K. A. , Ritchey, P. A. , & Cahoon, B. A. (2011). Survey and DNA Barcoding of Poaceae in Flat Rock Cedar Glades and Barrens State Natural Area, Murfreesboro, Tennessee. Castanea, 76(3), 300–310. https://doi.org/www.jstor.org/stable/41301505

[ece33891-bib-0016] Durham, S. R. , Walker, S. M. , Varga, E. M. , Jacobson, M. R. , O'Brien, F. , Noble, W. , … Nouri‐Aria, K. T. (1999). Long‐term clinical efficacy of grass‐pollen immunotherapy. The New England Journal of Medicine, 341, 468–475. https://doi.org/10.1056/nejm199908123410702 10441602 10.1056/NEJM199908123410702

[ece33891-bib-0017] Emberlin, J. (1997). Grass tree and weed pollens In Kay B. (Ed.) Allergy and allergic diseases (Vol. 2, pp. 845–857). Oxford: Blackwell Science.

[ece33891-bib-0018] Emberlin, J. , Jaeger, S. , Domínguez‐Vilches, E. , Galán‐Soldevilla, C. , Hodal, L. , Mandrioli, P. , … Bartlett, C. (2000). Temporal and geographical variations in grass pollen seasons in areas of western Europe: An analysis of season dates at sites of the European pollen information system. Aerobiologia, 16, 373–379. https://doi.org/10.1023/a:1026521331503

[ece33891-bib-0019] Erbas, B. , Akram, M. , Dharmage, S. C. , Tham, R. , Dennekamp, M. , Newbigin, E. , … Abramson, M. J. (2012). The role of seasonal grass pollen on childhood asthma emergency department presentations. Clinical & Experimental Allergy, 42(5), 799–805. https://doi.org/10.1111/j.1365-2222.2012.03995.x 22515396 10.1111/j.1365-2222.2012.03995.x

[ece33891-bib-0020] Feo Brito, F. , Mur Gimeno, P. , Carnés, J. , Fernández‐Caldas, E. , Lara, P. , Alonso, A. M. , … Guerra, F. (2010). Grass pollen, aeroallergens, and clinical symptoms in Ciudad Real, Spain. Journal of Investigational Allergology and Clinical Immunology, 20(4), 295–302. 20815307

[ece33891-bib-0021] Fernández Rodríguez, S. , Adams‐Groom, B. , Palacios, I. S. , Caeiro, E. , Brandao, R. , Ferro, R. , … Tormo Molina, R. (2014). Comparison of Poaceae pollen counts recorded at sites in Portugal, Spain and the UK. Aerobiologia, 31(1), 1–10. https://doi.org/10.1007/s10453-014-9338-2

[ece33891-bib-0022] Ferreira, F. , Hawranek, T. , Gruber, P. , Wopfner, N. , & Mari, A. (2004). Allergic cross‐reactivity: From gene to the clinic. Allergy, 59(3), 243–267. https://doi.org/10.1046/j.1398-9995.2003.00407.x 14982506 10.1046/j.1398-9995.2003.00407.x

[ece33891-bib-0023] Frenguelli, G. , Passalacqua, G. , Bonini, S. , Fiocchi, A. , Incorvaia, C. , Marcucci, F. , … Frati, F. (2010). Bridging allergologic and botanical knowledge in seasonal allergy: A role for phenology. Annals of Allergy, Asthma & Immunology, 105(3), 223–227. https://doi.org/10.1016/j.anai.2010.06.016 10.1016/j.anai.2010.06.016 20800789

[ece33891-bib-0024] Galán, C. , Cariñanos, P. , Alcázar, P. , & Domínguez, E. (2007). Spanish Aerobiology Network (REA): Management and quality manual. Córdoba, España: Universidad de Córdoba.

[ece33891-bib-0025] Galán, C. , Cuevas, J. , Infante, F. , & Dominguez, E. (1989). Seasonal and diurnal variation of pollen from Gramineae in the atmosphere of Cordoba (Spain). Allergologia et Immunopathologia, 17(5), 245–249. 2610187

[ece33891-bib-0026] Galán, C. , Emberlin, J. , Domínguez, E. , Bryant, R.H. , & Villamandos, F. (1995). A comparative analysis of daily variations in the Gramineae pollen counts at Córdoba, Spain and London, UK. Grana, 34(3), 189–198. https://doi.org/10.1080/00173139509429042

[ece33891-bib-0027] Galán, C. , Smith, M. , Thibaudon, M. , Frenguelli, G. , Oteros, J. , Gehrig, R. , … Brandao, R. (EAS Working Group ) (2014). Pollen monitoring: Minimum requirements and reproducibility of analysis. Aerobiologia, 30(4), 385–395. https://doi.org/10.1007/s10453-014-9335-5

[ece33891-bib-0028] Galimberti, A. , De Mattia, F. , Bruni, I. , Scaccabarozzi, D. , Sandionigi, A. , Barbuto, M. , … Labra, M. (2014). A DNA barcoding approach to characterize pollen collected by honeybees. PLoS One, 9(10), e109363 https://doi.org/10.1371/journal.pone.0109363 25296114 10.1371/journal.pone.0109363 PMC4190116

[ece33891-bib-0029] García‐Mozo, H. , Mestre, A. , & Galán, C. (2010). Phenological trends in southern Spain: A response to climate change. Agricultural and Forest Meteorology, 150(4), 575–580. https://doi.org/10.1016/j.agrformet.2010.01.023

[ece33891-bib-0030] Ghitarrini, S. , Galán, C. , Frenguelli, G. , & Tedeschini, E. (2017). Phenological analysis of grasses (Poaceae) as a support for the dissection of their pollen season in Perugia (Central Italy). Aerobiologia, 33(3), 339–349. https://doi.org/10.1007/s10453-017-9473-7

[ece33891-bib-0031] Ghitarrini, S. , Tedeschini, E. , Timorato, V. , & Frenguelli, G. (2017). Climate change: Consequences on the pollination of grasses in Perugia (Central Italy). A 33‐year‐long study. International Journal of Biometeorology, 61(1), 149–158. https://doi.org/10.1007/s00484-016-1198-8 27329325 10.1007/s00484-016-1198-8

[ece33891-bib-0032] Hawkins, J. , de Vere, N. , Griffith, A. , Ford, C.R. , Allainguillaume, J. , Hegarty, M. J. , … Adams‐Groom, B. (2015). Using DNA metabarcoding to identify the floral composition of honey: A new tool for investigating honey bee foraging preferences. PLoS One, 10(8), e0134735 https://doi.org/10.1371/journal.pone.0134735 26308362 10.1371/journal.pone.0134735 PMC4550469

[ece33891-bib-0033] Hirst, J. M. (1952). An automatic volumetric spore‐trap. Annals of Applied Biology, 39(2), 257–265. https://doi.org/10.1111/j.1744-7348.1952.tb00904.x

[ece33891-bib-0034] Hrabina, M. , Peltre, G. , Van Ree, R. , & Moingeon, P. (2008). Grass pollen allergens. Clinical & Experimental Allergy Reviews, 8(1), 7–17. https://doi.org/10.1111/j.1472-9733.2008.00126.x

[ece33891-bib-0035] Jäger, S. , Mandrioli, P. , Spieksma, F. , Emberlin, J. , Hjielmroos, M. , Rantio‐Lehtimaki, A. , … Ickovic, M. R. (1995). News. Aerobiologia, 11(1), 69–70. https://doi.org/10.1007/bf02136148

[ece33891-bib-0036] Jutel, M. , Jaeger, L. , Suck, R. , Meyer, H. , Fiebig, H. , & Cromwell, O. (2005). Allergen‐specific immunotherapy with recombinant grass pollen allergens. Journal of Allergy and Clinical Immunology, 116(3), 608–613. https://doi.org/10.1016/j.jaci.2005.06.004 16159631 10.1016/j.jaci.2005.06.004

[ece33891-bib-0037] Keller, A. , Danner, N. , Grimmer, G. , Ankebrand, M. , von der Ohe, K. , & von der Ohe, W. (2015). Evaluating multiplexed next‐generation sequencing as a method in palynology for mixed pollen samples. Plant Biology, 17(2), 558–566. https://doi.org/10.1111/plb.12251 25270225 10.1111/plb.12251

[ece33891-bib-0038] Kraaijeveld, K. , de Weger, L.A. , Ventayol García, M. , Buermans, H. , Frank, J. , & Hiemstra, P. S. (2015). Efficient and sensitive identification and quantification of airborne pollen using next‐generation DNA sequencing. Molecular Ecology Resources, 15(1), 8–16. https://doi.org/10.1111/1755-0998.12288 24893805 10.1111/1755-0998.12288

[ece33891-bib-0039] Laffer, S. , Spitzauer, S. , Susani, M. , Pairleitner, H. , Schweiger, C. , Grönlund, H. , … Valenta, R. (1996). Comparison of recombinant timothy grass pollen allergens with natural extract for diagnosis of grass pollen allergy in different populations. Journal of Allergy and Clinical Immunology, 98(3), 652–658. https://doi.org/10.1016/s0091-6749(96)70099-4 8828543 10.1016/s0091-6749(96)70099-4

[ece33891-bib-0040] Laube, I. , Hird, H. , Brodmann, P. , Ullmann, S. , Schӧne‐Michling, M. , Chrisholm, J. , & Broll, H. (2010). Development of primer and probe sets for the detection of plant species in honey. Food Chemistry, 118(4), 979–986. https://doi.org/10.1016/j.foodchem.2008.09.063

[ece33891-bib-0041] Leiferman, K. M. , & Gleich, G. J. (1976). The cross‐reactivity of IgE antibodies with pollen allergens. I. Analyses of various species of grass pollens. Journal of Allergy and Clinical Immunology, 58(1 pt. 2), 129–139. 956553 10.1016/0091-6749(76)90148-2

[ece33891-bib-0042] Lockey, R. F. , & Bukantz, S. C. (Eds.) (1998). Allergen immunotherapy (2nd ed.). New York, NY: Marcel Dekker.

[ece33891-bib-0043] Longhi, S. , Cristofori, A. , Gatto, P. , Cristofolini, F. , Grando, M. S. , & Gottardini, E. (2009). Biomolecular identification of allergenic pollen: A new perspective for aerobiological monitoring? Annals of Allergy, Asthma & Immunology, 103(6), 508–514. https://doi.org/10.1016/s1081-1206(10)60268-2 10.1016/S1081-1206(10)60268-2 20084845

[ece33891-bib-0044] Mabberley, D. J. (1987). The plant‐book: A portable dictionary of the higher plants. Cambridge: Cambridge University Press.

[ece33891-bib-0045] Mari, A. (2003). Skin test with a timothy grass (*Phleum pratense*) pollen extract vs. IgE to a timothy extract vs. IgE to rPhl p 1, rPhl p 2, nPhl p 4, rPhl p 5, rPhl p 6, rPhl p 7, rPhl p 11 and rPhl p12: Epidemiological and diagnostic data. Clinical & Experimental Allergy, 33(1), 43–51. https://doi.org/10.1046/j.1365-2222.2003.01569.x 12534548 10.1046/j.1365-2222.2003.01569.x

[ece33891-bib-0046] Mason‐Gamer, R. J. , Weil, C. F. , & Kellogg, E. A. (1998). Granule‐bound starch synthase: Structure, function, and phylogenetic utility. Molecular Biology and Evolution, 15(12), 1658–1673. 9866201 10.1093/oxfordjournals.molbev.a025893

[ece33891-bib-0047] Mohanty, R. P. , Buchheim, M. A. , & Levetin, E. (2017). Molecular approaches for the analysis of airborne pollen. A case study of Juniperus pollen. Annals of Allergy, Asthma & Immunology, 118(2), 204–211. https://doi.org/10.1016/j.anai.2016.11.015 10.1016/j.anai.2016.11.015 28024990

[ece33891-bib-0048] Moreira, P. F. , Gangl, K. , Vieira Fde, A. , Ynoue, L. H. , Linhart, B. , Flicker, S. , … Niederberger, V. (2015). Allergen microarray indicates Pooideae sensitization in Brazilian grass pollen allergic patients. PLoS One, 10(6), e0128402 https://doi.org/10.1371/journal.pone.0128402 26067084 10.1371/journal.pone.0128402 PMC4465745

[ece33891-bib-0049] Niederberger, V. , Laffer, S. , Fröschl, R. , Kraft, D. , Rumpold, H. , Kapiotis, S. , … Spitzauer, S. (1998). IgE antibodies to recombinant pollen allergens (Phl p 1, Phl p 2, Phl p 5, and Bet v 2) account for a high percentage of grass pollen‐specific IgE. Journal of Allergy and Clinical Immunology, 101(2), 258–64. https://doi.org/10.1016/s0091-6749(98)70391-4 9500760 10.1016/s0091-6749(98)70391-4

[ece33891-bib-0050] Nilsson, S. , & Persson, S. (1981). Tree pollen spectra in the Stockholm region (Sweden), 1973–1980. Grana, 20(3), 179–182. https://doi.org/10.1080/00173138109427661

[ece33891-bib-0051] Peltre, G. (2007). In: Immunologia molecolare degli allergeni di pollini di graminacee. Expression (Ed. Stallergens), 26, 8–12.

[ece33891-bib-0052] Perveen, A. (2006). A contribution to the pollen morphology of family Gramineae. World Applied Sciences Journal, 1(2), 60–65.

[ece33891-bib-0053] Plaza, M. P. , Alcázar, P. , Velasco‐Jiménez, M. J. , & Galán, C. (2017). Aeroallergens: A comparative study of two monitoring methods. Aerobiologia, 33(3), 363–373. https://doi.org/10.1007/s10453-017-9475-5

[ece33891-bib-0054] Richardson, R. T. , Lin, C. H. , Quijia, J. O. , Riusech, N. S. , Goodell, K. , & Johnson, R. M. (2015). Rank‐based characterization of pollen assemblages collected by honey bees using a multi‐locus metabarcoding approach. Applications in Plant Science, 3(11), apps. 1500043 https://doi.org/10.3732/apps.1500043 10.3732/apps.1500043 PMC4651628 26649264

[ece33891-bib-0055] Richardson, R. T. , Lin, C.‐H. , Sponsler, D. B. , Quijia, J. O. , Goodell, K. , & Johnson, R. M. (2015). Applications of ITS2 metabarcoding to determine the provenance of pollen by honeybees in an agrosystem. Applications in Plant Science, 3(1), piiapps. 1400066. https://doi.org/10.3732/apps.1400066 10.3732/apps.1400066 PMC4298230 25606352

[ece33891-bib-0056] Sánchez‐Mesa, J. A. , Smith, M. , Emberlin, J. , Allitt, U. , Caulton, E. , & Galán, C. (2003). Characteristics of grass pollen seasons in areas of southern Spain and the United Kingdom. Aerobiologia, 19(3‐4), 243–250. https://doi.org/10.1023/b:aero.0000006597.44452.a3

[ece33891-bib-0057] Sickel, W. , Ankenbrand, M. J. , Grimmer, G. , Holzschuh, A. , Härtel, S. , Lanzen, J. , … Keller, A. (2015). Increased efficiency in identifying mixed pollen samples by meta‐barcoding with a dual‐indexing approach. BMC Ecology, 15, 20 https://doi.org/10.1186/s12898-015-0051-y 26194794 10.1186/s12898-015-0051-y PMC4509727

[ece33891-bib-0058] Smith, M. , Emberlin, J. , Stach, A. , Rantio‐Lehtimäki, A. , Caulton, E. , Thibaudon, M. , … Galán, C. (2009). Influence of the North Atlantic Oscillation on grass pollen counts in Europe. Aerobiologia, 25(4), 321–332. https://doi.org/10.1007/s10453-009-9136-4

[ece33891-bib-0060] Tamura, K. , Stecher, G. , Peterson, D. , Filipski, A. , & Kumar, S. (2013). MEGA6: Molecular Evolutionary Genetics Analysis Version 6.0. Molecular Biology and Evolution, 30(12), 2725–2729. https://doi.org/10.1093/molbev/mst197 24132122 10.1093/molbev/mst197 PMC3840312

[ece33891-bib-0061] Tang, L. Y. , Nagata, N. , Matsushima, R. , Chen, Y. , Yoshioka, Y. , & Sakamoto, W. (2009). Visualization of plastids in pollen grains: Involvement of FtsZ1 in pollen plastid division. Plant Cell & Physiology, 50(4), 904–908. https://doi.org/10.1093/pcp/pcp042 10.1093/pcp/pcp042 19282372

[ece33891-bib-0062] Torricelli, M. , Pierboni, E. , Tovo, G. R. , Curcio, L. , & Rondini, C. (2016). In‐house validation of a DNA extraction protocol from honey and bee pollen and analysis in fast Real‐Time PCR of commercial honey samples using a knowledge‐based approach. Food Analytical Methods, 9(12), 3439–3450. https://doi.org/10.1007/s12161-016-0539-x

[ece33891-bib-0063] Tripodi, S. , Frediani, T. , Lucarelli, S. , Macrì, F. , Pingitore, G. , Di Rienzo Businco, A. , … Matricardi, P. M. (2012). Molecular profiles of IgE to *Phleum pratense* in children with grass pollen allergy: Implication for specific immunotherapy. Journal of Allergy and Clinical Immunology, 129(3), 834–839. https://doi.org/10.1016/j.jaci.2011.10.045 22206774 10.1016/j.jaci.2011.10.045

[ece33891-bib-0064] Untergrasser, A. , Cutcutache, I. , Koressaar, T. , Ye, J. , Faircloth, B. C. , Remm, M. , & Rozen, S. G. (2012). Primer3 — new capabilities and interfaces. Nucleic Acids Research, 40(15), e115 https://doi.org/10.1093/nar/gks596 22730293 10.1093/nar/gks596 PMC3424584

[ece33891-bib-0065] Valenta, R. , Lidholm, J. , Niederberger, V. , Hayek, B. , Kraft, B. , & Grӧlund, H. (1999). The recombinant allergen‐based concept of component‐resolved diagnostics and immunotherapy (CRD and CRIT). Clinical & Experimental Allergy, 29(7), 896–904. https://doi.org/10.1046/j.1365-2222.1999.00653.x 10383589 10.1046/j.1365-2222.1999.00653.x

[ece33891-bib-0066] Valenta, R. , Vrtala, S. , Ebner, C. , Kraft, D. , & Scheiner, O. (1992). Diagnosis of grass pollen allergy with recombinant Timothy grass (*Phleum pratense*) pollen allergens. International Archives of Allergy and Immunology, 97(4), 287–294. 1597349 10.1159/000236135

[ece33891-bib-0067] Valentini, A. , Miquel, C. , & Taberlet, P. (2010). DNA barcoding for honey biodiversity. Diversity, 2(4), 610–617. https://doi.org/10.3390/d2040610

[ece33891-bib-0068] Van Ree, R. , van Leeuwen, W. A. , & Aalberse, R. C. (1998). How far can we simplify in vitro diagnostics for grass pollen allergy?: A study with 17 whole pollen extracts and purified natural and recombinant major allergens. Journal of Allergy and Clinical Immunology, 102(2), 184–90. https://doi.org/10.1016/s0091-6749(98)70084-3 9723659 10.1016/s0091-6749(98)70084-3

[ece33891-bib-0069] Waiblinger, H. U. , & Grohmann, L. (2014). Guidelines for validation of DNA extraction methods applied in subsequent PCR analysis of food and feed products for the presence of genetically modified material. Journal für Verbraucherschutz und Lebensmittelsicherheit, 9, 183–190. https://doi.org/10.1007/s00003-014-0862-3

[ece33891-bib-0070] Weber, R.W. (2004). Cross‐reactivity of pollen allergens. Current Allergy and Asthma Reports, 4(5), 401–408. https://doi.org/10.1007/s11882-004-0091-4 15283881 10.1007/s11882-004-0091-4

[ece33891-bib-0072] Zhang, Y. , Bielory, L. , Mi, Z. , Cai, T. , Robock, A. , & Georgopoulos, P. (2015). Allergenic pollen season variations in the past two decades under changing climate in the United States. Global Change Biology, 21(4), 1581–1589. https://doi.org/10.1111/gcb.12755 25266307 10.1111/gcb.12755 PMC4356643

