# Supplementary material for: New biomolecular tools for aerobiological monitoring: Identification of major allergenic Poaceae species through fast real‐time PCR
Source: Ecol Evol. 2018 Mar 25;8(8):3996–4010. doi: 10.1002/ece3.3891 (PMC5916283; doi:10.1002/ece3.3891)
Supplement: Supplementary file 1 [file ECE3-8-3996-s001.docx]

Supporting table 1. After applying the purification kit to DNA extracted from melinex and *D. glomerata* loose pollen (DG), Cqs to *actin* increase or no amplification is present at all. Und = Undetermined, no amplification.

|  |  | Fast PCR real-time for *actin* gene | | | |
| --- | --- | --- | --- | --- | --- |
| Sample | Purification kit | Cq | mean Cq | st. dev | ∆Cq |
|  |  |  |  |  |  |
| 30A  (April 30^th^) |  | 37.70 | 37.32 | 0.54 | 1.23* |
|  |  | 36.93 |  |  |  |
|  | x | Und |  |  |  |
|  |  | Und |  |  |  |
| 07M  (May 7^th^) |  | 36.12 | 36.33 | 0.30 | 2.02 |
|  |  | 36.54 |  |  |  |
|  | x | 39.98 |  |  |  |
|  |  | Und |  |  |  |
| 09M  (May 9^th^) |  | 34.16 | 34.25 | 0.12 | 1.92 |
|  |  | 34.33 |  |  |  |
|  | x | 37.20 | 37.62 | 0.58 | 1.84 |
|  |  | 38.03 |  |  |  |
| pollen | | | | | |
| **DG** |  | 27.17 | 27.36 | 0.26 | 1.69 |
|  |  | 27.55 |  |  |  |
| **DG**  **1^st^ elution** | x | 27.98 | 27.94 | 0.07 | 2.31 |
|  |  | 27.89 |  |  |  |
| **DG**  **2^nd^ elution** |  | 29.10 | 29.22 | 0.18 | 1.81 |
|  |  | 29.35 |  |  |  |

*low quality DNA

Supporting table 2. Results of SYBR Green chemistry test: amplification is present also when using a non-target species as template. The difference between melting temperatures (T_m_) of the target species and the other ones (Δ) are small or null, suggesting that SYBR Green is not suitable for single species detection with our primers.

| **SYBR Green** | | | | | | | | | | | | | | | | | | | | | |
| --- | --- | --- | --- | --- | --- | --- | --- | --- | --- | --- | --- | --- | --- | --- | --- | --- | --- | --- | --- | --- | --- |
| SYSTEM | **Da 1** | | | **Ph 1** | | | **Ph 2** | | | **Lo 1** | | | **Fe 1** | | | **Poa 1** | | | **Poa 2** | | |
| **TARGET SPECIES** | ***D. glomerata*** | | | ***P. pratense*** | | | | | | ***L. perenne*** | | | ***F. arundinacea*** | | | ***P. pratensis*** | | | | | |
| PRIMER FOR | Da matK 4-F | | | Ph matK 1-F | | | Da matK 1-F | | | Lo matK 1-F | | | Fe matK 2-F | | | Poa matK 1-F | | | Poa matK 3-F | | |
| PRIMER REV | Ph matK 1-R | | | Ph matK 1-R | | | Ph matK 3-R | | | Lo matK 1-R | | | Ph matK 1-R | | | Poa matK 1-R | | | Poa matK 3-R | | |
| **Template↓** | **Cq** | **T_m_** | **Δ** | **Cq** | **T_m_** | **Δ** | **Cq** | **T_m_** | **Δ** | **Cq** | **T_m_** | **Δ** | **Cq** | **T_m_** | **Δ** | **Cq** | **T_m_** | **Δ** | **Cq** | **T_m_** | **Δ** |
| *D. glomerata* | **15.64** | **77.9** |  | 25.16 | 76.9 | 0 | 27.54 | 75.1 | 0 | 26.02 | 75.7 | -0.3 | 39.16 | 77.3 | 0.1 | 34.35 | 77.9 | 0.4 | 23.48 | 75.1 | -1.2 |
| *P. pratense* | 17.74 | 76.6 | -1.3 | **14.22** | **76.9** |  | **16.90** | **75.1** |  | 27.77 | 75.7 | -0.3 | 34.70 | 77.2 | 0 | 23.44 | 77.2 | -0.3 | 19.31 | 76.6 | 0.3 |
| *L. perenne* | 14.63 | 76.9 | -1 | 23.00 | 77.2 | 0.3 | 24.86 | 75.1 | 0 | **14.54** | **76** |  | 28.91 | 77.2 | 0 | 28.49 | 77.5 | 0 | 19.96 | 75.1 | -1.2 |
| *F. arundinacea* | 14.59 | 76.9 | -1 | 23.92 | 77.2 | 0.3 | 25.11 | 75.3 | 0.2 | 18.67 | 76 | 0 | **15.29** | **77.2** |  | 28.77 | 77.5 | 0 | 19.95 | 74.8 | -1.5 |
| *P. pratensis* | 18.64 | 76.9 | -1 | 19.57 | 77.5 | 0.6 | 19.38 | 74.7 | -0.4 | 28.03 | 75.9 | -0.1 | 33.63 | 77.5 | 0.3 | **15.77** | **77.5** |  | **14.42** | **76.3** |  |
| c- (water) | Und | 61.7 | -16 | Und | 61.7 | -15 | Und | 72 | -3.1 | Und | 75.3 | -0.7 | Und | 61.7 | -16 | Und | 71.4 | -6.1 | 36.39 | 72.9 | -3.4 |

Supporting table 3. A. Da1 was the first system tested for *D. glomerata*. Using an annealing temperature (T_ann_) of 60°C, amplification was observed also in non-target species, with low Cq differences (Δ). Da1, and two new combinations, Da2 and Da3, were then tested with T_ann_ of 65°C. No specificity was obtained, but Da2 was further applied raising T_ann_ gradually. With a T_ann_ of 67°C. amplification was obtained only in reactions with *D. glomerata*. B, C, D. Single primer sets were assessed for *P. pratense*. *F. arundinacea* and *P. Pratensis*, Ph1, Fe1 and Poa1, respectively. Fast real-time PCRs were repeated at raising T_ann_ until specific amplification was observed.

4A

| **TaqMan® matK-PGP probe** | | | | | | | | | | | | |
| --- | --- | --- | --- | --- | --- | --- | --- | --- | --- | --- | --- | --- |
| *species* | ***D. glomerata*** | | | | | | | | | | | |
| SYSTEM | **Da 1** | | **Da 1** | | **Da 2** | | **Da 3** | | **Da 2** | | | |
| PRIMER FOR | Da matK 4-F | | Da matK 4-F | | Da matK 4-F | | Da matK 1-F | | Da matK 4-F | | | |
| PRIMER REV | Ph matK 1-R | | Ph matK1-R | | Da matK 1-R | | Da matK 1-R | | Da matK 1-R | | | |
| T_ann_ | **60°C** | | **65°C** | | | | | | 66°C | | **67°C** | |
| **Template↓** | **Cq** | Δ | **Cq** | Δ | **Cq** | Δ | **Cq** | Δ | **Cq** | Δ | **Cq** | Δ |
| *D. glomerata* | **16.80** |  | **16.26** |  | **17.46** |  | **17.68** |  | **24.56** |  | **27.88** |  |
| *P. pratense* | 20.02 | 3.22 | 23.26 | 7 | 31.17 | **13.7** | 26.87 | 9.19 | 37.02 | 12.5 | Und |  |
| *L. perenne* | 17.64 | 0.84 | 17.26 | 1 | 25.38 | **7.92** | 25.56 | 7.88 | 32.22 | 7.66 | Und |  |
| *F. arundinacea* | 17.58 | 0.78 | 16.9 | 0.63 | 25.34 | **7.88** | 25.55 | 7.87 | 31.6 | 7.04 | Und |  |
| *P. pratensis* | 20.64 | 3.84 | 24.45 | 8.19 | 29.17 | **11.7** | 28.59 | 10.9 | 34.99 | 10.4 | Und |  |
| c- (water) | Und |  | Und |  | Und |  | Und |  | Und |  | Und |  |

4B

| **TaqMan® matK-PGP probe** | | | | | | | | |
| --- | --- | --- | --- | --- | --- | --- | --- | --- |
| *species* | ***P. pratense*** | | | | | | | |
| SYSTEM | **Ph 1** | | | | | | | |
| PRIMER FOR | Ph matK 1-F | | | | | | | |
| PRIMER REV | Ph matK 1-R | | | | | | | |
| T_ann_ | **60°C** | | **65°C** | | **66°C** | | **67°C** | |
| **Template↓** | **Cq** | Δ | **Cq** | Δ | **Cq** | Δ | **Cq** | Δ |
| *D. glomerata* | 24.83 | 8.76 | 26.17 | 10.2 | Und |  | Und |  |
| *P. pratense* | **16.07** |  | **15.95** |  | **16.43** |  | **27.63** |  |
| *L. perenne* | 25.34 | 9.26 | 25.98 | 10 | 27.92 | 11.5 | Und |  |
| *F. arundinacea* | 25.84 | 9.77 | 26.27 | 10.3 | 27.56 | 11.1 | Und |  |
| *P. pratensis* | 19.39 | 3.31 | 25.41 | 9.46 | 29.4 | 13 | Und |  |
| c- (water) | Und |  | Und |  | Und |  | Und |  |

4C

| **TaqMan® matK-PGP probe** | | | | | | |
| --- | --- | --- | --- | --- | --- | --- |
| *species* | ***F. arundinacea*** | | | | | |
| SYSTEM | **Fe 1** | | | | | |
| PRIMER FOR | Fe matK 2-F | | | | | |
| PRIMER REV | Ph matK 1-R | | | | | |
| T_ann_ | **60°C** | | **65°C** | | **66°C** | |
| **Template↓** | **Cq** | Δ | **Cq** | Δ | **Cq** | Δ |
| *D. glomerata* | 36.46 | 19.74 | Und |  | Und |  |
| *P. pratense* | 35.85 | 19.13 | Und |  | Und |  |
| *L. perenne* | 23.14 | 6.42 | 36.97 | 15.4 | Und |  |
| *F. arundinacea* | **16.72** |  | **21.54** |  | **27.85** |  |
| *P. pratensis* | 34.29 | 17.57 | Und |  | Und |  |
| c- (water) | Und |  | Und |  | Und |  |

4D

| **TaqMan® matK-PGP probe** | | | | | | |
| --- | --- | --- | --- | --- | --- | --- |
| *species* | ***P. pratensis*** | | | | | |
| SYSTEM | **Poa 1** | | | | | |
| PRIMER FOR | Poa matK 1-F | | | | | |
| PRIMER REV | Poa matK 1-R | | | | | |
| T_ann_ | **60°C** | | **65°C** | | **66°C** | |
| **Template↓** | **Cq** | Δ | **Cq** | Δ | **Cq** | Δ |
| *D. glomerata* | 31.98 | 15.99 | Und |  | Und |  |
| *P. pratense* | 27.17 | 11.18 | Und |  | Und |  |
| *L. perenne* | 29.60 | 13.61 | 35.54 | 15.8 | Und |  |
| *F. arundinacea* | 29.80 | 13.81 | 37.64 | 18 | Und |  |
| *P. pratensis* | **15.99** |  | **19.69** |  | **25.35** |  |
| c- (water) | Und |  | Und |  | Und |  |

Supporting table 4. Sensitivity of species-specific systems was assessed in fast real-time PCR reactions containing progressively lower template amount (two replicates per dilution). Da2 and Ph1 showed the highest detection capacity, amplifying when at least 0.15 ng of template are present (0.038 ng and 0.019 ng are not considered valid for Ph1 because ̴2 Cqs of difference between the two replicates indicate low reproducibility). Sensitivity is lower for Fe1 (0.6 ng) and Poa1 (6 ng).

| dilution | Template  ng/reaction | **Da 2** | | **Ph 1** | | **Fe 1** | | **Poa 1** | |
| --- | --- | --- | --- | --- | --- | --- | --- | --- | --- |
| 1/10 | **6** | 29.33 | 29.75 | 28.68 | 28.56 | 32.86 | 31.95 | **37** | **38.03** |
| 1/10 | **0.6** | 34.41 | 34.51 | 32.14 | 32.07 | **37.54** | **37.84** | Und | Und |
| 1/4 | **0.15** | **37.52** | **38.15** | **36.2** | **36.57** | Und | Und | Und | Und |
| 1/4 | 0.038 | Und | Und | 39.33 | 37.36 | Und | Und | Und | Und |
| 1/2 | 0.019 | Und | Und | 38.27 | 36.74 | Und | Und | Und | Und |
| 1/2 | 0.009 | Und | Und | 37.85 | Und | Und | Und | Und | Und |
| 1/2 | 0.005 | Und | Und | Und | Und | Und | Und | Und | Und |
| 1/2 | 0.002 | Und | Und | Und | Und | Und | Und | Und | Und |
| 1/2 | 0.001 | Und | Und | Und | Und | Und | Und | Und | Und |

Supporting figure 1. Alignment of *matK* sequences from the five species of interest. Position of the designed primers and matK-PGP probe are evidenced.
