# Supplementary material for: New biomolecular tools for aerobiological monitoring: Identification of major allergenic Poaceae species through fast real‐time PCR
Source: Ecol Evol. 2018 Mar 25;8(8):3996–4010. doi: 10.1002/ece3.3891 (PMC5916283; doi:10.1002/ece3.3891)
Supplement: Supplementary file 2 [file ECE3-8-3996-s002.pdf]

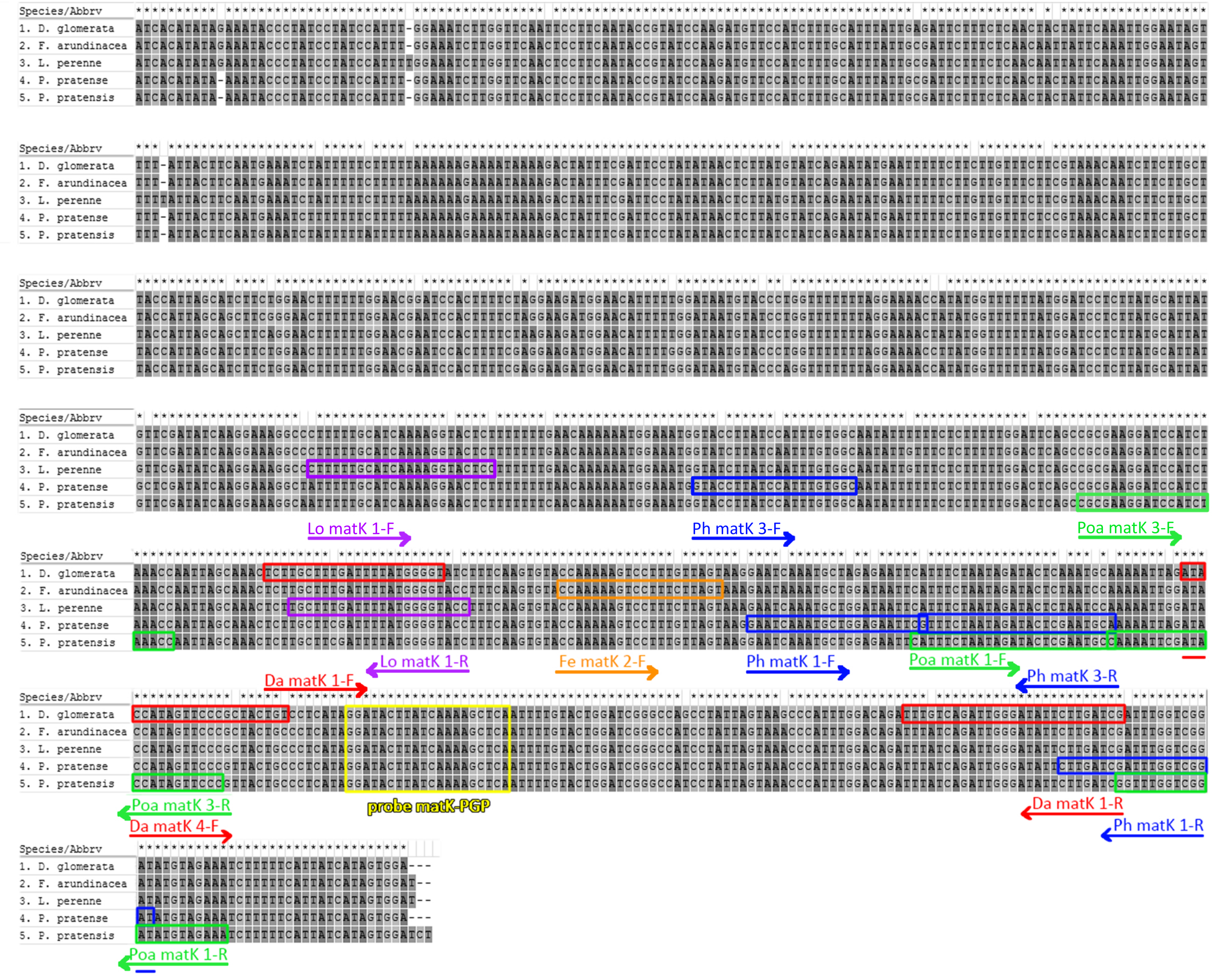
Species/Abbrv
1. D. glomerata
2. F. arundinacea
3. L. perenne
4. P. pratense
5. P. pratensis
Lo matK 1-F
Ph matK 3-F
Poa matK 3-F
Lo matK 1-R
Fe matK 2-F
Ph matK 1-F
Poa matK 1-F
Ph matK 3-R
Da matK 1-F
Poa matK 3-R
probe matK-PGP
Da matK 1-R
Da matK 4-F
Ph matK 1-R
Poa matK 1-R
